# Prevalence of and factors associated with unmanageable pain levels in people with knee or hip osteoarthritis: a cross-sectional population-based study

**DOI:** 10.1186/s12891-022-06110-1

**Published:** 2023-01-23

**Authors:** Daniela Costa, Eduardo B. Cruz, David G. Lopes, Catarina Nunes da Silva, Ana Rita Henriques, Diogo Luis, Jaime Branco, Helena Canhão, Ana M. Rodrigues

**Affiliations:** 1 NOVA National School of Public Health, Public Health Research Centre, Universidade NOVA de Lisboa, Lisbon, Portugal; 2 Comprehensive Health Research Centre (CHRC), Universidade Nova de Lisboa, Lisbon, Portugal; 3 EpiDoC Unit, NOVA Medical School (NMS), Universidade NOVA de Lisboa, Lisbon, Portugal; 4 Physiotherapy Department, School of Health, Polytechnic Institute of Setúbal, Setúbal, Portugal; 5 Serviço de Reumatologia do Hospital Egas Moniz, Centro Hospital Lisboa Ocidental (CHLO), Lisbon, Portugal; 6 Rheumatology Unit, Hospital dos Lusíadas, Lisbon, Portugal

**Keywords:** Osteoarthritis, Pain management, Pharmacological therapies, Non-pharmacological therapies

## Abstract

**Background:**

Pain due to knee and / or hip osteoarthritis (HKOA) is the most common symptom for seeking healthcare. Pain interferes on daily activities, social and occupational participation in people with HKOA. The goal of this study is to estimate the prevalence of unmanageable pain levels (UPL) among people with HKOA), characterize this population and identify factors associated with UPL, and compare therapeutic strategies used by people with UPL versus manageable pain levels (MPL).

**Methods:**

We analysed data from the EpiReumaPt study (*n* = 10,661), that included a representative sample of the Portuguese population. Among these, 1081 participants had a validated diagnosis of HKOA by a rheumatologist.. Sociodemographic, lifestyle and health-related data were collected in a structured interview. Pain intensity (NPRS) data were collected in a medical appointment. Painmedication (last month), physiotherapy and surgery were considered as therapies for pain management. UPL was defined as a mean pain intensity in the previous week of ≥5 points on 11-point numeric pain rating scale. The factors associated with UPL were analyzed with logistic regression (*p* < 0.05, 95%CI). The effect of unmanageable pain levels was assessed by the HOOS/KOOS activities of daily living and quality of life subscales. Symptoms of anxiety and depression were assessed by the Hospital Anxiety and Depression Scale (HADS). Analysis was completed with linear and logistic regression. All analysis were weighted.

**Results:**

The estimated prevalence of UPL among people with HKOA was 68.8%. UPL was associated with being female (odds ratio (OR) = 2.36, *p* < 0.001), being overweight (OR = 1.84, *p* = 0.035) or obese (OR = 2.26, *p* = 0.006), and having multimorbidity (OR = 2.08, *p* = 0.002). People with UPL reported worse performance in activities of daily living and lower quality of life (β = − 21.28, *p* < 0.001 and β = − 21.19, p < 0.001, respectively) than people with MPL. People with UPL consumed more NSAIDs (22.0%, *p* = 0.003), opioids (4.8%, *p* = 0.008), paracetamol (2.7%, *p* = 0.033), and overall analgesics (7.3%, *p* = 0.013) than people with MPL. A higher proportion of people with UPL underwent physiotherapy (17.5%, *p* = 0.002) than people with MPL.

**Conclusion:**

Two-thirds of people with HKOA in Portugal have poor management of their pain levels. Clinical and lifestyle factors, that are highly presented in individuals with HKOA, are associated with unmanageable pain. Our results highlighting the need for further research and implementation of effective interventions to improve pain, function and quality of life in people with HKOA.

**Supplementary Information:**

The online version contains supplementary material available at 10.1186/s12891-022-06110-1.

## Introduction

Osteoarthritis (OA) is the most common joint disease, affecting more than 300 million people worldwide. The hip and knee joints are the most affected and combined reflect 9.6 million years lived with disability [1]. The direct annual costs of hip and/or knee OA (HKOA) per patient are estimated at 6.7 K€ worldwide, which reaches 10.8 K€ if total joint replacement surgery is considered [2]. Indirect annual costs per patient are estimated between 0.2 K–12.3 K€. In Portugal, indirect costs represent 0.4% of the national gross domestic product [3]. Moreover, the incidence of total joint replacement surgery in Portugal has increased by 20% annually in the period between1990–2011, which represents the highest growth rate among Organisation for Economic Co-operation and Development countries [4].

HKOA often results with chronic pain, physical disability, and mental health and sleep problems, which impairs quality of life (QoL) and prevents participation in social and occupational activities [5]. Pain is the most disabling symptom of OA and a major driver of clinical decision-making and healthcare resources [5].

Pain severity is more indicative of functional impairment than radiographic severity [6–8]. Pain severity is also strongly associated with reported disability, medication use [9, 10], healthcare utilization, impact on daily and occupational activities, loss of productivity, early retirement, and absenteeism [11, 12]. Poor pain management is a major predictor of total joint replacement surgery [13].

Recent literature raises concerns over the pain management and low QoL among people with HKOA [10, 14]. Current pain management focus on symptom control that incorporates medication as the first-line intervention increases the risk of opioid prescription [14, 15]. The use of end-stage interventions such as surgery is becoming more common, even among people with early-stage OA [14, 15]. The knowledge of pain management and therapeutic strategies in Portugal is scarce. There is a critical need to better understand patients’ associated risk factors, characteristics, and available therapeutic interventions for people enduring HKOA in Portugal. Therefore, the aims of this study were to: 1) estimate the prevalence of unmanageable pain levels (UPL) among Portuguese population with HKOA, 2) characterize the HKOA population in terms of sociodemographic, lifestyle, and health-related variables and identify factors associated with UPL; and 3) compare performance in activities of daily living (ADL), QoL, anxiety and depression symptoms, and therapies used between people with UPL versus people with manageable pain levels (MPL). This knowledge will help to understand which factors undermine patient’s ability to manage pain levels and how traditional therapies used either facilitate or fail to facilitate successful management of pain. The results of this study may also indicate whether the associated therapeutic interventions account for the factors associated with poor pain management and if improvements in offered interventions to people with HKOA are needed.

## Methods

### Data source

We analysed data from the EpiReumaPt, a national cross-sectional, population based study with a representative sample of the Portuguese population that aimed to investigate the burden of rheumatic and musculoskeletal diseases (RMDs) in Portugal. As described in detail elsewhere [16], participant recruitment was conducted between September 2011 and December 2013 using a random selection of private households in Portugal stratified by administrative territorial units (NUTS II: Norte, Centro, Lisboa and Vale do Tejo, Alentejo, Algarve, Azores, and Madeira) and the size of the population within each locality. In each household, the permantent resident ≥18 years old who most recently celebrated birthday was selected to participate in the study. In total, 28,502 households were contacted, 8041 individuals refused to participate, and 10,661 were included in the study. The EpiReumaPt population was similar to the Portuguese population (Census 2011) in age strata, sex, and NUTII distribution [16].

EpireumaPt data collection was performed using a three-staged approach. In the first stage, participants completed a face-to-face interview to collect sociodemographic and health-related information and to screen for RMDs. Interviews were conducted by a team of non-medical healthcare professionals trained for this purpose, and data were collected using a computer-assisted personal interview system. Screening was considered positive if a participant mentioned a previously known RMD, if any algorithm in the screening questionnaires was positive, or if the participant reported muscle, vertebral, or peripheral joint pain in the previous 4 weeks.

In the second stage, participants who screened positive for at least one RMD (*n* = 7451) and approximately 20% of participants who screened negative for RMDs (*n* = 701) were invited to a clinical appointment at the primary care center of the participant’s neighborhood. Participants were seen by a multidisciplinary team consisting of a rheumatologist, X-ray technician, and nurse. Clinical assessment consisted of a structured evaluation, laboratory tests, and imaging exams, if needed, to establish a diagnosis and evaluate disease-related information. According to participants’ complaints, simple radiographs were performed in 122 hips and 479 knees, among other joints. Rheumatologists were blind to prior health-related data. Of the participants in the second stage, 4275 did not attend the clinical appointment. Therefore, at the end of the second stage, there were 3877 clinical observations: 3198 participants received an RMD diagnosis, and 679 did not receive an RMD diagnosis.

**Fig. 1:**
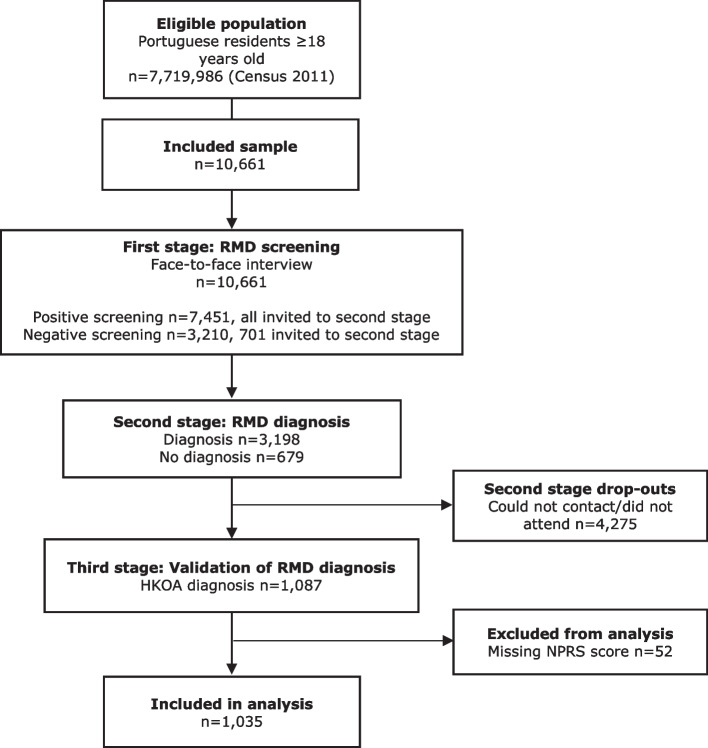
EpiReumaPt study flowchart

In the third stage, three experienced rheumatologists reviewed all data and validated the RMD diagnosis of individuals that participated in the clinical appointment – second stage. Diagnostic agreement among the three rheumatologists was 98.3%, with a Cohen’s K coefficient of 10.87 (95% confidence interval (CI): 0.83, 0.91) [16]. When data were insufficient to fulfill international classification criteria for an RMD, five rheumatologists met to reach agreement on the final diagnosis. When doubts persisted, the opinion of the rheumatologist who performed the clinical assessment in the second stage prevailed. From the individuals that participated in the clinical appointment (*n* = 3.877), a total of 1087 participants had a validated diagnosis of HKOA, 199 had a validated diagnosis of hip OA, and 981 had a validated diagnosis of knee OA (Fig. 1). When the overall performance of the RDM screening algorithm was evaluated, using final diagnosis after the third stage as the gold standard, its sensitivity and specificity were 98 and 22% and positive and negative predictive value were 85 and 71%, respectively.

### Study population

This study included participants of EpiReumaPt with validated a diagnosis of HKOA according to American College of Rheumatology criteria [17, 18].

### Case definition and measurement

Mean pain intensity in the previous week, measured on a 11-point numeric pain rating scale (NPRS) in the second stage of EpiReumaPt, was used to categorize participants with HKOA into MPL (< 5 points) and UPL (≥5 points), which was validated by Zelman et al. (2003) using the question 5 of Brief Pain Inventory (BPI) scale, as the average pain in the previous week on an 11-point NPRS. The optimal cut-off point found for manageable day pain in OA was 5 [F(7, 90)= 7.08, *p*<0.0001] [19]. In the validation of this cut-off Zelman et al., statistically derived the 11-point NPRS with BPI interference scale measures, WOMAC-physical function and SF-36 and reach the final cut off of 5 in average pain intensity that best separate the groups with manageable pain and not manageable pain. This cut-off represent a “Manageable Day” or, in other words, in people with HKOA, pain intensity less than 5 on a 11-point NPRS permit an increased function and quality of life [19].

When both the hip and knee were affected, the worst score was considered.

### Sociodemographic, clinical, and lifestyle variables

Sociodemographic, clinical, and lifestyle variables were collected during the first and second phases of EpiReumaPt. To assure better clinical interpretation, some variables were subjected to categorical transformation.

#### Sociodemographic and anthropometric variables

Sociodemographic variables were age, sex, and geographic location according to NUTS II territorial units. Madeira and Azores were merged in the analysis as the Islands region. Marital status was categorized as “partner” (married or consensual union) or “no partner” (single, widowed, or divorced). Education level was categorized according to years of education completed: < 4 years (less than primary education), 4–9 years (primary or secondary education), or ≥ 10 years (secondary or higher education).

Body mass index (BMI) was categorized as underweight (≤18.49 kg/m^2^), healthy weight (≥18.50 and ≤ 24.99 kg/m^2^), overweight (≥25.00 and ≤ 29.99 kg/m^2^), or obese (≥30.00 kg/m^2^).

#### Lifestyle and clinical variables

Lifestyle variables were alcohol intake (“no” or “occasionally or daily”), smoking habits (“never” and “occasionally or daily”), and regular exercise/sports (“yes” or “no”).

The number of chronic non-communicable diseases was calculated as the numeric count of the following self-reported conditions: high blood pressure, high cholesterol, cardiac disease, diabetes mellitus, chronic lung disease, problems in the digestive tract, renal colic, neurological disease, allergies, mental or psychiatric illness, cancer, thyroid or parathyroid problems, hypogonadism, and hyperuricemia. Multimorbidity was defined as having two or more chronic non-communicable diseases [20].

In addition to pain intensity, other clinical variables were considered: performance in ADL, QoL, and the presence of depression and/or anxiety symptoms. Performance in ADL and QoL related to HKOA were evaluated with the Portuguese version of the Knee Injury and Osteoarthritis Outcome Scale (KOOS) [21] and Hip Disability and Osteoarthritis Outcome Scale (HOOS) [22]. These self-reported clinical outcome measures evaluate short- and long-term consequences of HKOA in five dimensions: pain, symptoms, ADL, sports and leisure, and QoL. For this study, we used only the HOOS/KOOS ADL and HOOS/KOOS QoL subscales. Scores for each dimension were transformed on a 0–100 scale, with 0 representing extreme hip/knee problems and 100 representing no hip/knee problems [21, 22]. For both subscales, if more than one joint was affected, the worst score was considered.

Anxiety and depression symptoms were evaluated using the Hospital Anxiety and Depression Scale subscales for depression (HADS-D) and anxiety (HADS-A). Both scales have a range of 0 to 21, with higher values representing more severe symptoms of anxiety or depression. Final HADS-A and HADS-D scores were categorized using validated cut-offs as: “with anxiety” (HADS-A ≥ 11) or “without anxiety” (HADS-A < 11) and “with depression” (HADS-D ≥ 11) or “without depression” (HADS-D < 11) [23].

#### Use of therapies

Information on pharmacological therapies, defined as daily medications taken in the previous month, was collected in the first-stage interviews. Medication for pain relief was classified according to the Anatomical Therapeutic Chemical Classification System as: glucosamine (M01AX05); analgesics/antipyretics (N02B), specifically paracetamol (N02BE01); simple (N02A) and combined (N02AJ) opioids; non-steroidal anti-inflammatory drugs (NSAIDs; M01A); and topical agents (M02A). Information on physiotherapy attendance in the previous 12 months, was also collected in the first-stage interviews. Information on any previous hip or knee surgery was collected during the second-stage clinical appointments, which occurred no more than 15 days after the first stage. Surgery variable (yes/no) was related to any hip or knee surgical procedure that the participants have had in their life, this may include, for example, replacement surgery, meniscectomy or ligament reconstruction surgery.

### Data analysis

After participants were categorized into UPL and MPL subgroups, weighted proportions of participants with UPL were computed taking sampling design into account as described in the EpiReumaPt methodologic protocol [16]. The logit transformation method was used to calculate 95% CIs. Analysis of the proportion of participants with UPL and MPL (relative and absolute frequencies) was conducted separately for participants with hip OA and those with knee OA.

Descriptive statistics were used to characterize all participants and separate, the MPL and UPL subgroups, according to sociodemographic, lifestyle, health-related variables as well as use of pain medication, physiotherapy and surgery. Differences between subgroups were analyzed using independent samples t-tests for continuous variables and Chi-square tests for categorical variables.We first analyzed associations between sociodemographic, lifestyle, and health-related variables and pain levels subgroups. Variables with *p* < 0.25 were included in a univariate logistic regression model in a forward selection process [24] to avoid early exclusion of potentially important variables (Additional file 1). These variables were selected in this stage since they are previously known associated with HKOA outcomes [25]. Variables with *p* < 0.05 were then kept in a backward selection process to construct a multivariable model [26].

We next analyzed associations between UPL and clinical outcomes, having MPL subgroup as reference. Associations between UPL and HOOS/KOOS ADL and QoL subscale scores were analyzed using linear regression models adjusted for the variables retained in the multivariable model. Associations between UPL and the presence of anxiety and depression symptoms were analyzed using logistic regression models adjusted for the same variables. This adjustment was made since the variables retained in the first multivariate model showed statistical associations with the outcome. The adjustment analyzed any confounder effect on the association between UPL/MPL with other clinical outcomes.

Given the scarcity of data, normal and underweight BMI categories were merged into a single category (< 25.00 kg/m^2^). A sensitivity analysis was additionally performed for participants with hip OA and for those with knee OA (Additional file 2).

All analyses were weighted and performed with SPSS 26 complex samples for MacOS (IBM Corp., Armonk, NY, USA). Statistical significance was defined as *p* < 0.05.

## Results

The prevalence of UPL among people with HKOA was 68.8%. People with UPL reported a mean pain intensity of 6.85 ± 1.54 on a 0- to 10-point numeric pain rating scale. The proportions of people with UPL who had hip OA (*n* = 144, 69.7%) or knee OA (*n* = 694, 69.5%) were similar (Table 1).

**Table 1:** Prevalence of UPL and MPL among people with HKOA

	Total	UPL	MPL
		(NPRS ≥ 5)	(NPRS < 5)
**HKOA weighted prevalence**	100%	68.8% (63.9, 73.2)	31.2% (26.8, 36.1)
**(95% CI)**	*n* = 1035	*n* = 765	*n* = 270
**Sample size**			
**HKOA weighted count**	1,080,633	743,130	337,502
**Pain (NPRS), mean ± SD**	5.55 ± 2.45	6.85 ± 1.54	2.69 ± 1.43
**Hip OA, n (%)**	199 (2.9)	144 (69.7)	40 (30.3)
**Knee OA, n (%)**	981 (12.4)	694 (69.5)	247 (30.4)

All percentages and means ± SDs are weighted

MPL, Manageable pain levels; CI, confidence interval; HKOA, hip and knee osteoarthritis; UPL, unmanageable pain levels; NPRS, numeric pain rating scale; OA, osteoarthritis; SD, standard deviation

**Fig. 2:**
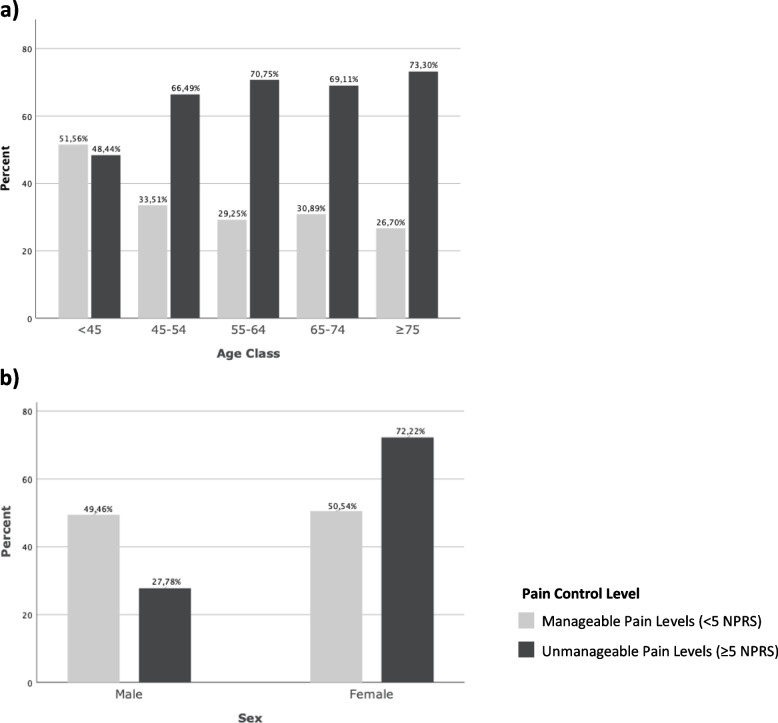
Proportions of people with HKOA and UPL or MPL by (**a**) age class (years) and (**b**) sex

The proportion of people with UPL increased with age, reaching 73.3% in the oldest age class (≥75 years of age). UPL was more common in female than in male subjects, as shown in Fig. 2.

Mean age and age class distributions were similar between UPL and MPL subgroups (Table 2). The UPL subgroup contained a larger proportion of people with a lower level of education (< 4 years of completed schooling) than the MPL subgroup. Overweight and obesity were highly prevalent among people with HKOA and were present at similar proportions between UPL and MPL subgroups.

**Table 2:** Sociodemographic, lifestyle, and health-related variables for people with HKOA

	Total *n* = 1035	UPL *n* = 765	MPL *n* = 270	*p*-value^a^
Age (mean ± SD)	64.33 ± 12.90	65.32 ± 12.04	62.17 ± 14.38	0.091
< 45 years, *n* (%)	38 (6.1)	24 (4.3)	14 (10.2)	0.265
45–54 years, *n* (%)	138 (6.0)	98 (15.7)	40 (17.4)	
55–64 years, *n* (%)	279 (23.2)	209 (23.9)	70 (21.7)	
65–74 years, *n* (%)	351 (31.3)	261 (31.4)	90 (30.9)	
≥75 years, *n* (%)	229 (23.2)	173 (24.7)	56 (19.8)	
Female sex, *n* (%)	744 (65.4)	571 (72.2)	173 (50.5)	< 0.001
Geographic location, *n* (%)				0.478
North	281 (35.6)	205 (36.0)	76 (34.8)	
Centre	255 (27.8)	180 (26.2)	75 (21.4)	
Lisbon	171 (23.7)	121 (23.4)	50 (24.4)	
Alentejo	69 (6.4)	53 (7.0)	16 (5.1)	
Algarve	22 (1.9)	18 (2.3)	4 (1.1)	
Islands	237 (4.6)	188 (5.3)	49 (3.3)	
Marital status, *n* (%)				
With partner	662 (63.8)	477 (64.0)	185 (63.3)	0.893
Years of education, *n* (%)				0.024
< 4 years	257 (23.0)	208 (26.6)	49 (15.1)	
4–9 years	652 (62.2)	474 (58.9)	178 (68.9)	
≥10 years	124 (14.8)	82 (14.2)	42 (16.0)	
BMI, *n* (%)				0.067
Underweight/normal weight	173 (21.1)	114 (18)	59 (27.8)	
Overweight	404 (43.4)	294 (43.5)	110 (43.3)	
Obese	381 (35.4)	297 (38.5)	84 (28.8)	
Lifestyle factors, *n* (%)				
Smoker	75 (10.9)	47 (7.9)	28 (17.3)	0.015
Daily alcohol intake	225 (28.5)	152 (24.5)	73 (37.1)	0.016
Regular exercise	220 (21.5)	146 (19.4)	74 (26.2)	0.116
Multimorbidity, *n* (%)	783 (73.8)	608 (79.4)	175 (61.5)	< 0.001

All percentages and means±SDs are weighted

^a^p-value from independent samples t-tests for continuous variables and Chi-square tests for categorical variables. Significance level (p < 0.05) based on adjusted F

*MPL* Manageable pain levels, *BMI* body mass index, *HKOA* hip and knee osteoarthritis, *UPL* unmanageable pain levels, *SD* standard deviation

The UPL subgroup contained smaller proportions of people who smoked or consumed alcohol daily than the MPL subgroup. Although regular exercise was more common within the MPL subgroup than within the UPL subgroup, this difference was not significant. The presence of multimorbidity was more common within the UPL subgroup than within the MPL subgroup.

When analyzing associations between UPL and sociodemographic, lifestyle, and health-related variables, female participants, being overweight or obese, and having multimorbidity were independently associated with UPL (Table 3).

**Table 3:** Multivariable model including factors associated with UPL in people with HKOA

	UPL vs. MPL OR (95% CI)	p-value
Variables included		
Female sex^a^	2.32 (1.50 to 3.57)	< 0.001
BMI^b^		
Overweight	1.84 (1.04 to 3.25)	0.035
Obese	2.26 (1.27 to 4.02)	0.006
Multimorbidity^c^	2.07 (1.33 to 3.20)	0.001

*n* = 1009. ^a^Reference class: male sex; ^b^Reference class: underweight/normal weight; ^c^Reference class: no multimorbidity. Wald F(5)=8.08, *p* < 0.001; Nagelkerke Pseudo R^2^ = 0.125.MPL manageable pain levels, *BMI* body mass index, *CI* confidence interval, *HKOA* hip and knee osteoarthritis, *UPL* unmanageable pain levels, *OR* odds ratio

When adjusting for sex, BMI, and multimorbidity, significant negative associations between UPL and HOOS/KOOS ADL and QoL scores remained (Table 4). Thus, people with UPL were more likely to have worse HOOS/KOOS ADL and QoL scores than people with MPL. Although there was a significant association between UPL and anxiety symptoms in the unadjusted model, no significant association was found in the adjusted model, having the MPL subgroup as reference. No statistically significant associations were found in the unadjusted or in the adjusted model between depression symptoms and UPL, having MPL subgroup as reference. No differences were found in the sensitivity analysis (Additional file 2).

**Table 4:** HOOS/KOOS ADL and QoL subscale scores and anxiety and depression symptoms in people with HKOA and UPL or MPL

	Total	UPL	MPL		Crude β (95% CI)	*p*-value	Adjusted β (95% CI)	*p*-value
Sample size (n) and weighted prevalence (%)	*n* = 1035 (100%)	*n* = 765 (68.4%)	*n* = 270 (31.6%)					
HOOS/KOOS	66.00	58.36	83.06	UPL	−24.70	< 0.001	−21.28	< 0.001
ADL, mean ± SD	±23.08	±21.31	±16.96		(−28.60, −20.80)		(−24.81, −17.76)	
HOOS/KOOS	50.55	43.09	67.00	UPL	−23.91	< 0.001	−21.19	< 0.001
QoL, mean ± SD	±22.45	±19.37	±19.88		(−28.31, −19.51)		(−25.22, −17.16)	
					**Crude OR ** **(95% CI)**	***p*-value**	**Adjusted OR ** **(95% CI)**	***p*-value**
Anxiety	198	161	37 (12.8)	UPL	1.76	0.020	1.23	0.395
symptoms, n (%)	(18.1)	(20.6)			(1.09, 2.83)		(0.77, 1.97)	
Depression	162	130	32 (12.9)	UPL	1.49	0.235	1.11	0.744
symptoms, n (%)	(16.5)	(18.1)			(0.77, 2.86)		(0.59, 2.12)	

All percentages and means±SDs are weighted. **β** and OR are adjusted for sex, obesity, and multimorbidity

*ADL* activities of daily living, *MPL* manageable pain levels, *CI* confidence interval, *HKOA* hip and knee osteoarthritis, *HOOS* Hip Disability and Osteoarthritis Outcome Scale, *UPL* unmanageable pain levels, *KOOS* Knee Injury and Osteoarthritis Outcome Scale, *OR* odds ratio, *QoL* quality of life, *SD* standard deviation

Overall, NSAIDs, analgesics, and physiotherapy were the most used therapies by people with HKOA (Table 5). Higher proportions of people within the UPL subgroup regularly took NSAIDS, simple opioids, and analgesics, specifically paracetamol, than within the MPL subgroup. Physiotherapy was also more commonly used by people with UPL than by people with MPL. There were no significant differences in the proportions of people who underwent hip or knee surgery between UPL and MPL subgroups.

**Table 5:** Pharmacological, conservative non-pharmacological, and surgical therapies used by people with HKOA with UPL or MPL

	Total	UPL	MPL	p-value^a^
Sample size and weighted prevalence n(%)	*n* = 1035 (100)	*n* = 765 (68.4)	*N* = 270 (31.6)	
Pharmacological therapies				
*Anti-inflammatory*				
NSAIDS, n (%)	239 (19.0)	194 (22.0)	45 (12.5)	0.003
Topical NSAIDS, n (%)	10 (1.2)	7 (1.5)	3 (0.7)	0.410
*Opioids*				
Simple opioids, n (%)	49 (3.7)	44 (4.8)	5 (1.3)	0.008
Opioids combined with analgesics, n (%)	24 (1.4)	22 (1.8)	2 (0.4)	0.053
*Analgesics/antipyretics*				
Analgesics (all), n (%)	72 (5.7)	62 (7.3)	10 (2.7)	0.013
Paracetamol, n (%)	25 (2.0)	23 (2.7)	2 (0.6)	0.033
*Others*				
Glucosamine, n (%)	65 (4.7)	53 (5.1)	12 (3.9)	0.438
*Conservative non-pharmacological therapies*				
Physiotherapy, n (%)	152 (14.9)	121 (17.5)	31 (8.9)	0.002
*Surgery*				
Hip surgery^b^, n (%)	42 (18.7)	29 (19.1)	13 (17.6)	0.847
Knee surgery^c^, n (%)	113 (13.2)	82 (13.4)	31 (13.0)	0.893

All percentages are weighted

^a^*p*-value from Chi-square tests. Significance level is based on adjusted F. ^b^Sub-sample with hip OA. ^c^Sub-sample with knee OA

*MPL* manageable pain levels, *CI* confidence interval, *HKOA* hip and knee osteoarthritis, UPL, Unmanageable pain levels, *NSAIDS* non-steroidal anti-inflammatory drugs, *OA* osteoarthritis

## Discussion

Our results indicate that 68.8% of people with HKOA in Portugal live with UPL, which is higher than the prevalence of UPL in Mexico (53%) [27], the Survey of Osteoarthritis Real World Therapies (SORT) cohort from six European countries (54%) [10], and a sample of Portuguese people with knee OA included in the SORT cohort (51%) [28]. All three of these earlier studies included people who were ≥ 50 years old, possibly excluding younger patients with early OA, and who took analgesics regularly, which may explain why we found a higher prevalence of UPL in the present study.Our study included a representative sample of the Portuguese population who live in the community, suggesting that offered interventions do not meet the need for pain control for more than two-thirds of the Portuguese HKOA population.

We found that people with UPL had lower education levels than people with MPL. This finding is consistent with previous literature reporting that low education is associated with more severe OA symptoms, is a social determinant of unhealthy lifestyles and multimorbidity [27, 29], is a determinant of lack of access to and delay in seeking healthcare [30], and is associated with increased pain intensity over time [31].

The multivariable model showed that female sex, overweight and obesity, and multimorbidity were associated with UPL, similar to the results of the SORT study [10]. In our study being female was associated with higher OA-related pain levels, but gender differences on pain intensity in HKOA remain unclear in the literature. Data from quantitative sensory testing in people with knee OA has shown that women have greater sensitivity to pain but no sex differences were found in clinical pain. These authors suggested that women have an enhanced central pain sensitivity [30]. More recently, Mun et al. (2020) concluded that women with knee OA have a greater interleukin 6 activation when compared to men, after laboratory quantitative sensory testing. These authors concluded that this enhanced inflammatory reactivity in women may contribute to exacerbation or maintenance of symptoms [31].

Other factors like psychosocial and genetic factors showed inconsistent gender differences and pain severity [32] [33].. Also, a systematic review of progression phenotypes among people with OA shows that overweight or obesity is a major factor in the progression of OA and is associated with worsening of pain, loss of physical function, and structural deterioration over time [34].

Additionally, our results show that having multimorbidity was associated with UPL. Multimorbidity is associated with chronic pain in a cumulative manner [35] and is related to pain intensity in people with HKOA [36]. People with multimorbidity have a higher likelihood of walking impairments, which can contribute to a worsening of OA and other chronic conditions that occurswith an additional consequence of psychological distress [35, 36].

We found that UPL was negatively associated with performance in ADL and QoL. Previous research reveals that within the OA population, pain severity explains most of the variability in disability and QoL [37]. High pain severity may lead to fear of movement and/or avoidance behaviors, resulting in physical inactivity and less participation in social activities and leads to greater physical disability, psychological distress, and reduced QoL [38].

The sensitivity analysis did not show differences when separating participants with hip OA and with knee OA. This suggests that similar factors were associated with UPL in people with OA in these two joints, separately or together. However, due to the small sample size of participants with hip OA, we were not able to perform multivariable analysis for anxiety and depression symptoms.International clinical practice guidelines recommend that topical NSAIDs be considered before oral NSAIDs consistentwith the least systemic exposure principle, and oral NSAIDs are strongly recommended at the lowest possible dose [39]. Given the limited efficacy of paracetamol and its potentially harmful secondary effects, it is only conditionally recommended for people with OA [39]. Although tramadol is conditionally recommended, non-tramadol opioids are not recommended for the management of pain in people with OA. Tramadol and non-tramadol opioids can be used only when alternatives have been exhausted. Glucosamine is strongly not recommended for people with HKOA [39]. In the present study, oral NSAIDs were the most used medication followed by analgesics/antipyretic medication, whereas topical NSAIDs were the least used pharmacological modality. People with UPL regularly took more medication for pain relief, namely NSAIDS, opioids, and analgesics, specifically paracetamol, than people with MPL, consistent with the results of the SORT study [10]. A cohort study from the Netherlands also shows pain severity is positively related to analgesic intake; however, the authors concluded that most reasons for analgesic prescription are unknown [40].

Even though no temporal relationships can be drawn from a cross-sectional design, this study reveals that a higher proportion of people who took daily pain medication in the previous month had UPL. Additionally, our results suggest that medication is taken by a much lower proportion of people with OA in Portugal than in other European countries [12]. (12)In Europe, medication use seems to be highly variable across countries. Data from five countries included in the National Health and Wellness Survey (*n* = 37,650), reveal that the minimum and maximum proportions of overall pain medication intake by people with OA was 22% in Germany and 53.2% in Spain. Specifically, NSAID’s were at minimum by 46,5% of people with OA in France and at maximum by 81.9% in Germany; Paracetamol was not used by any participants of Germany, but by 6% of participants from Spain. Opioids were used by 1.8% in Italy at minimum and by 54.5% at maximum of people with HKOA in France. These proportions of medication use were much higher than the ones seen in our findings probably because medication intake was asked as “medication used at the moment” [12] rather than daily use of medication in the previous month, as in our study. Similarly, our findings reveal that NSAID’s are the medication most used by people with HKOA. In other European countries opioids are the second most used pain medication, contrarily to our study (used similarly to analgesics). These findings may suggest that opioids are less prescribed in Portugal than in other countries. However, data from the general population reveal that the prescription of opioids in Portugal has increased by 1.5-fold between 2013 and 2017 [41].

Although randomized controlled trials show that analgesic drugs and other recommended interventions effectively manage pain in individuals with OA, adherence to medication and healthy lifestyle behaviors are a real-world concern that prevent the optimization of pain control in this population. A qualitative meta-ethnographic study points out that factors such as the severity of pain, perceived effect of medication, fear related to side effects, acceptability of dose regimens, education and knowledge about OA and the medication regimen, self-efficacy, and locus of control over OA influence medication adherence [42].

Regarding conservative non-pharmacological therapies used by people with HKOA, we found that < 20% of people with UPL underwent physiotherapy in the last 12 months or regularly exercised. These values are much lower than other European countries. For example, the proportion of people with OA in national cohorts that used physiotherapy in a 12-month time frame was 53.1% in Germany [43] and 32% in the UK [44].

Crrent clinical guidelines recommend physiotherapy and exercise as first-line treatments. Exercise should be maintained during the progression of the disease for pain management purposes [45, 46]. Although, similarly to our results, current literature suggests that exercise and physiotherapy, as part of core non-pharmacological treatments, are uptake by < 50% of people with HKOA [47, 48]. Additionally, healthcare interventions seems to be symptom-driven and segmented [49] centered on pharmacological [47, 48] and surgical options [50]. On the other hand, lack of long-term adherence to behavior-dependent interventions, like exercise has been pointed out as an explanation for poor long-term outcomes in people with HKOA [51, 52]. Therefore, interventions for the management of HKOA should also target behavior changethrough strategies that optimize motivation and overcome barriers of adherence [53].

### Limitations

Our study has several limitations that should be considered. A large proportion of participants included in the first phase of the study and invited for the second phase did not show up in the rheumatologist appointment. Therefore, we should hypothesize a selection bias, since the subjects who were willing to participate in the appointment might be the most severe cases.

Due to its cross-sectional design, no cause-effect relationships can be established between UPL and sociodemographic, lifestyle, and health-related variables. Additionally, identification of variables with direct and indirect effect on the outcome is not possible with this study design and was not accounted in the regression models [54]. Also, giving the cross-sectional design of the study, the variables related with the use of therapies were considered only to describe and compare UPL/MPL subgroups and no associations with the outcome variables were explored. Also, estimation of the proportion of people with UPL in the Portuguese population using sample weights is not free from error, although sample weights are recommended for all statistical analyses using complex samples data [55].

[1, 43](58)We used the validated cut-off by Zelman et al. (2003), to define people with UPL and MPL. However, in this validation study the eligible criteria included people with HKOA that have at least 1 year pain duration, that have a daily use of some form of analgesic and that reported average daily pain of 4 at least in the 11-point NPRS [19]. Notwithstanding, the cut-off “5” was previously validated in other populations as pain intensity that interferes with function and QoL - musculoskeletal pain in general [56], in patients with knee OA [10] and in those with HKOA waiting for surgery [57]. In this late study the authors concluded that the interference of pain in function (using the same cut-off) was independent from pain duration. Therefore, we cautiously believe that this cut-off is valid also in the population of our study.

We asked participants about their use of “regular medication”. However, as people with OA often use analgesic medication sporadically for pain flares rather than daily, our results may underestimate the proportion of people that use medication for symptomatic control. On the other hand, we did not control for other pain conditions like fibromyalgia or widespread pain syndromes, pain duration or pain frequency (e.g. daily or constant pain) that may increase the intake of medication. Also, as physiotherapy attendance in the last 12 months was self-reported, we acknowledge the possibility that memory bias may compromise the accuracy of our results. Additionally, we did not investigate the reason for medication use or physiotherapy.

Surgery procedure was not specified, and this variable (surgery - yes/no) was not related to a specific time frame. Additionally, since the causes of surgery are not known our results were purposively descriptive and no hypothesis can be drawn. “Regular exercise” was self-reported by participants and did not consider the precise amount and intensity. Hence, our data may overestimate the proportion of people who exercised. Moreover, pain intensity is multifactorial [38], and several potentially important factors were not considered in the analysis, such as fear avoidance beliefs, catastrophizing, or coping strategies.

The analyzed data were collected in 2011–2013, but due to few specific strategies directed to RMDs in the last decade in Portugal, we cautiously believe that the current management of OA does not differ from that reflected in this study.

### Strengths and implications

This is the first population-based study in Portugal analyzing outcomes of current interventions offered to community-dwelling people with HKOA. The results of this study raise concerns regarding important factors that should be further explored in future research and addressed in national health policies to optimize the outcomes of people with HKOA, namely:

1) The high proportion of people with UPL, suggest a poor control of pain levels in people with HKOA;

2) The high proportion of people with UPL who use pharmacological and non-pharmacological therapiesmay indicate that pain management is suboptimal;

3) Besides the low proportion of people who use therapy, the interventions offered do not seem to be aligned with international recommendations [39, 45] considering the small proportion of people who underwent physiotherapy, exercised, and used pain medication and the large proportion of people who were overweight or obese.

## Conclusion

Approximately two-thirds of the Portuguese population with HKOA have UPL, despite the higher use of medication and physiotherapy in the UPL subgroup, when compared with theMPL subgroup. Being overweight and having multimorbidity are modifiable risk factors associated with UPL. Overall, recommended management strategies appear to be offered to a small proportion of people with HKOA. Our results may seemingly suggest that current therapies are sub-optimal and that lifestyle behaviour change may be neglected, but longitudinal research is needed to corroborate these hypotheses. Our findings reveal an opportunity for pain management improvement in the HKOA population and highlight the need for further research on effective pain management interventions.

## Supplementary Information

**Additional file 1.** Univariate Logistic Regression analysis. Table S1. Factors associated with unmanageable pain levels in univariate logistic regression analysis.

**Additional file 2.** Sensitivity Analysis of participants with hip OA and with knee OA. Table S.2 a). Sociodemographic, lifestyle, and health-related variables for people with Hip OA. Table S.2 b) Sociodemographic, lifestyle, and health-related variables for people with Knee OA. Table S.3- Univariable models factors associated with UPL in people with Knee OA or Hip OA. Table S.4- Multivariable models factors associated with UPL in people with Knee OA or Hip OA. Table S.5. HOOS/KOOS ADL and QoL subscale scores and anxiety and depression symptoms in people with Hip OA and UPL or MPL and people with Knee OA and UPL or MPL.

## Data Availability

The data underlying this article were provided by the EpiDoc Unit - CEDOC by permission. Data will be shared upon request to the corresponding author with the permission of EpiDoc Unit group leaders.

## Abbreviations

ADL: Activities of Daily Living
BMI: Body Mass Index
CI: Confidence Interval
HADS-A: Hospital Anxiety and Depression Scale subscale for anxiety
HADS-D: Hospital Anxiety and Depression Scale subscale for depression
HKOA: Hip and/or Knee Osteoarthritis
HOOS: Hip Disability and Osteoarthritis Outcome Scale
KOOS: Knee Injury and Osteoarthritis Outcome Scale
MPL: Manageable pain levels
NPRS: Numerical Pain Rating Scale
NSAIDs: Non-steroidal anti-inflammatory drugs
OA: Osteoarthritis
OR: Odds Ratio
QoL: Quality of Life
RMD: Rhematic and Musculoskeletal Diseases
SD: Standard Deviation
SORT: Survey of Osteoarthritis Real World Therapies
UPL: Unmanageable Pain Levels
