# Supplementary material for: Prevalence of and factors associated with unmanageable pain levels in people with knee or hip osteoarthritis: a cross-sectional population-based study
Source: BMC Musculoskelet Disord. 2023 Jan 23;24:60. doi: 10.1186/s12891-022-06110-1 (PMC9869512; doi:10.1186/s12891-022-06110-1)
Supplement: Supplementary file 1 — Additional file 1. Univariate Logistic Regression analysis. Table S1. Factors associated with unmanageable pain levels in univariate logistic regression analysis. [file 12891_2022_6110_MOESM1_ESM.docx]

**Additional File 1**

Univariate Logistic Regression analysis

| Table S1. Factors associated with unmanageable pain levels in univariate logistic regression analysis | | |
| --- | --- | --- |
|  | OR (95% CI) | p-value |
| Age class |  | 0.378 |
| <45 years old | 0.34 (0.12, 0.99) | 0.047 |
| 45-54 years old | 0.72 (0.34, 1.54) | 0.399 |
| 55-64 years old | 0.88 (0.47, 1.66) | 0.694 |
| 65-74 years old | 0.82 (0.47, 1.40) | 0.459 |
| ≥75 years old^a^ | - | - |
| Sex | |  |
| Male^a^ | - | - |
| Female | 2.54 (1.64, 3.96) | <0.001 |
| Chronic non-communicable diseases |  |  |
| No multimorbiditiy^a^ | - | - |
| Multimorbidity | 2.41 (1.49, 3.91) | <0.001 |
| Geographic location |  | 0.178 |
| North^a^ | - | - |
| Center | 0.81 (0.46, 1.39) | 0.440 |
| Lisbon | 0.93 (0.51, 1.69) | 0.802 |
| Alentejo | 1.33 (0.67, 2.65) | 0.410 |
| Algarve | 2.06 (0.64, 6.58) | 0.225 |
| Islands | 1.53 (0.90, 2.61) | 0.115 |
| Marital status |  |  |
| Without partner^a^ | - | - |
| With partner | 1.03 (0.64, 1.66) | 0.893 |
| Education level |  | 0.005 |
| <4 years^a^ | - | - |
| 4-9 years | 0.49 (0.31, 0.76) | 0.002 |
| ≥10 years | 0.49 (0.31, 0.76) | 0.038 |
| BMI (kg‎/m^2^) |  | 0.022 |
| Normal or underweight (<25kg/m^2^)^a^ |  |  |
| Overweight (25-29.99 kg‎/m^2^) | 1.96 (1.08, 3.57) | 0.028 |
| Obese (≥30 kg‎/m^2^) | 2.36 (1.28, 4.38) | 0.006 |
| Smoking habits |  |  |
| Non-smoker^a^ | - | - |
| Daily or occasional smoker | 0.41 (0.20, 0.85) | 0.017 |
| Alcohol consumption |  |  |
| Never or occasionally^a^ | - | - |
| Daily | 0.60 (0.39, 0.93) | 0.022 |
| Exercise |  |  |
| No regular exercise^a^ | - | - |
| Regular exercise | 0.67 (0.41, 1.10) | 0.117 |
| ^a^Reference classes  All analyses were weighted. | | |
