# Supplementary material for: Prevalence of and factors associated with unmanageable pain levels in people with knee or hip osteoarthritis: a cross-sectional population-based study
Source: BMC Musculoskelet Disord. 2023 Jan 23;24:60. doi: 10.1186/s12891-022-06110-1 (PMC9869512; doi:10.1186/s12891-022-06110-1)
Supplement: Supplementary file 2 — Additional file 2. Sensitivity Analysis of participants with hip OA and with knee OA. Table S.2 a). Sociodemographic, lifestyle, and health-related variables for people with Hip OA. Table S.2 b) Sociodemographic, lifestyle, and health-related variables for people with Knee OA. Table S.3- Univariable models factors associated with UPL in people with Knee OA or Hip OA. Table S.4- Multivariable models factors associated with UPL in people with Knee OA or Hip OA. Table S.5. HOOS/KOOS ADL and QoL subscale scores and anxiety and depression symptoms in people with Hip OA and UPL or MPL and people with Knee OA and UPL or MPL. [file 12891_2022_6110_MOESM2_ESM.docx]

**Additional file 2**

**Sensitivity Analysis of participants with hip OA and with knee OA**

**Table S.2 a).** Sociodemographic, lifestyle, and health-related variables for people with Hip OA

|  | Total  n=184 | UPL  n=144 | MPL  n=40 | *p*-value^a^ |  |
| --- | --- | --- | --- | --- | --- |
| Age (mean±SD) | 64.1±12.7 | 66.1±12.0 | 59.5±13.2 | 0.066 |  |
| <45 years, n (%) | 9 (6.5) | 7 (6.3) | 2 (7.1) | 0.490 |  |
| 45-54 years, n (%) | 19 (16.7) | 15 (12.7) | 4 (25.7) |  |  |
| 55-64 years, n (%) | 33 (21.5) | 27 (19.7) | 6 (25.7) |  |  |
| 65-74 years, n (%) | 73 (31.3) | 53 (31.4) | 20 (31.3) |  |  |
| ≥75 years, n (%) | 50 (23.9) | 42 (29.9) | 8 (10.1) |  |  |
| Female sex, n (%) | 114 (55.2) | 92 (66.3) | 22 (29.8) | 0.003 |  |
| Geographic location, n (%) |  |  |  | 0.166 |  |
| North | 55 (37.5) | 43 (36.0) | 12 (40.8) |  |  |
| Centre | 47 (30.3) | 33 (24.2) | 14 (44.4) |  |  |
| Lisbon | 13 (5.7) | 10 (6.2) | 3 (4.8) |  |  |
| Alentejo | 3 (1.6) | 3 (2.3) | - |  |  |
| Algarve | 28 (2.9) | 23 (3.5) | 5 (1.3) |  |  |
| Islands | 38 (22.0) | 32 (27.7) | 6 (8.7) |  |  |
| Marital status, n (%) |  |  |  |  |  |
| With partner | 123 (67.7) | 95 (69.4) | 28 (63.6) | 0.709 |  |
| Years of education, n (%) |  |  |  | 0.061 |  |
| <4 years | 46 (19.5) | 41 (24.7) | 5 (7.4) |  |  |
| 4-9 years | 113 (70.1) | 86 (65.0) | 27 (81.9) |  |  |
| ≥10 years | 24 (10.4) | 16 (10.3) | 8 (10.6) |  |  |
| BMI, n (%) |  |  |  | 0.466 |  |
| Underweight/normal weight | 34 (26.9) | 26 (24.8) | 8 (31.5) |  |  |
| Overweight | 70 (42.1) | 51 (38.7) | 19 (49.7) |  |  |
| Obese | 65 (31.0) | 55 (36.5) | 10 (18.7) |  |  |
| Lifestyle factors, n (%) |  |  |  |  | |
| Smoker | 9 (10.7) | 7 (6.1) | 2 (21.3) | 0.154 | |
| Daily alcohol intake | 55 (34.4) | 41 (28.8) | 14 (47.4) | 0.224 | |
| Regular exercise | 38 (18.0) | 25 (17.2) | 13 (19.8) | 0.751 | |
| Multimorbidity, n (%) | 138 (72.3) | 116 (81.4) | 22 (51.5) | 0.030 | |
| All percentages and means±SDs are weighted.  ^a^p-value from independent samples t-tests for continuous variables and Chi-square tests for categorical variables. Significance level (p<0.05) based on adjusted F.  MPL, Manageable pain levels; BMI, body mass index; UPL, unmanageable pain levels; SD, standard deviation | | | | | |

**Table S.2 b)** Sociodemographic, lifestyle, and health-related variables for people with Knee OA

|  | Total  n=941 | UPL  n=694 | MPL  n=247 | *p*-value^a^ |  |
| --- | --- | --- | --- | --- | --- |
| Age (mean±SD) | 64.6±12.9 | 65.4±11.9 | 62.8±14.9 | 0.093 |  |
| <45 years, n (%) | 31 (6.1) | 18 (3.7) | 13 (11.7) | 0.039 |  |
| 45-54 years, n (%) | 127 (15.1) | 90 (15.3) | 37 (14.5) |  |  |
| 55-64 years, n (%) | 263 (23.5) | 198 (25.3) | 65 (19.6) |  |  |
| 65-74 years, n (%) | 314 (31.5) | 234 (31.3) | 80 (32.0) |  |  |
| ≥75 years, n (%) | 206 (23.7) | 154 (24.4) | 52 (22.2) |  |  |
| Female sex, n (%) | 691 (67.9) | 531 (73.7) | 160 (54.5) | <0.001 |  |
| Geographic location, n (%) |  |  |  | 0.620 |  |
| North | 250 (34.4) | 182 (35.2) | 68 (32.5) |  |  |
| Centre | 253 (27.7) | 165 (26.8) | 68 (29.9) |  |  |
| Lisbon | 61 (6.5) | 46 (6.9) | 15 (5.5) |  |  |
| Alentejo | 19 (1.8) | 15 (2.0) | 4 (1.2) |  |  |
| Algarve | 224 (4.9) | 179 (5.4) | 45 (3.6) |  |  |
| Islands | 154 (24.8) | 107 (23.7) | 47 (27.3) |  |  |
| Marital status, n (%) |  |  |  |  |  |
| With partner | 597 (63.6) | 428 (63.4) | 169 (64.1) | 0.902 |  |
| Years of education, n (%) |  |  |  | 0.110 |  |
| <4 years | 237 (23.8) | 189 (26.6) | 48 (17.2) |  |  |
| 4-9 years | 593 (60.9) | 431 (58.7) | 162 (66.0) |  |  |
| ≥10 years | 110 (15.3) | 74 (14.7) | 36 (16.8) |  |  |
| BMI, n (%) |  |  |  | 0.144 |  |
| Underweight/normal weight | 149 (19.6) | 97 (16.9) | 52 (25.5) |  |  |
| Overweight | 369 (43.5) | 270 (44.1) | 99 (42.1) |  |  |
| Obese | 353 (36.9) | 273 (39.0) | 80 (32.4) |  |  |
| Lifestyle factors, n (%) |  |  |  |  | |
| Smoker | 69 (10.3) | 43 (8.1) | 26 (15.2) | 0.042 | |
| Daily alcohol intake | 193 (26.8) | 128 (23.0) | 65 (35.5) | 0.011 | |
| Regular exercise | 194 (21.5) | 130 (19.1) | 64 (27.0) | 0.090 | |
| Multimorbidity, n (%) | 718 (74.7) | 556 (80.0) | 162 (62.7) | 0.0004 | |
| All percentages and means±SDs are weighted.  ^a^p-value from independent samples t-tests for continuous variables and Chi-square tests for categorical variables. Significance level (p<0.05) based on adjusted F.  MPL, Manageable pain levels; BMI, body mass index; UPL, unmanageable pain levels; SD, standard deviation | | | | | |

**Table S.3-** Univariable models factors associated with UPL in people with Knee OA or Hip OA

|  | Hip OA |  | Knee OA |  |  |  |
| --- | --- | --- | --- | --- | --- | --- |
|  | UPL vs. MPL  OR (95% CI) | p-value | UPL vs. MPL  OR (95% CI) | p-value |  |  |
| Female sex^a^ | 4.64 (1.63; 13.19) | 0.004 | 2.34 (1.51; 3.62) | <0.001 |  |  |
| Age group^b^ |  |  |  |  |  |  |
| <45 years | 1.77 (0.11; 27.73) | 0.683 | 0.30 (0.10; 0.91) | 0.034 |  |  |
| 55-64 years | 1.54 (0.14; 16.51) | 0.719 | 1.22 (0.67; 2.22) | 0.513 |  |  |
| 65-74 years | 2.02 (0.33; 12.51) | 0.447 | 0.93 (0.51; 1.70) | 0.810 |  |  |
| ≥75 years | 5.97 (0.87; 41.20) | 0.070 | 1.04 (0.53; 2.03) | 0.904 |  |  |
| Geographic location^c^ |  |  |  |  |  |  |
| North | 0.68 (0.12; 3.79) | 0.659 | 0.87 (0.44; 1.75) | 0.704 |  |  |
| Centre | 0.42 (0.08; 2.22) | 0.305 | 0.72 (0.35; 1.47) | 0.368 |  |  |
| Alentejo | 1 | - | 1.31 (0.37; 4.66) | 0.679 |  |  |
| Algarve | 2.11 (0.39; 11.36) | 0.383 | 1.23 (0.60; 2.52) | 0.580 |  |  |
| Islands | 2.44 (0.49; 12.31) | 0.277 | 0.70 (0.32; 1.53) | 0.373 |  |  |
| Marital status^d^ |  |  |  |  |  |  |
| With partner | 1.30 (0.33; 5.13) | 0.709 | 0.97 (0.61; 1.55) | 0.902 |  |  |
| Years of education^e^ |  |  |  |  |  |  |
| 4-9 years | 0.24 (0.07; 0.82) | 0.023 | 0.58 (0.37; 0.89) | 0.014 |  |  |
| ≥10 years | 0.29 (0.07; 1.13) | 0.075 | 0.57 (0.28; 1.13) | 0.107 |  |  |
| BMI^f^ |  |  |  |  |  |  |
| Overweight | 0.99 (0.18; 5.44) | 0.989 | 1.58 (0.86; 2.90) | 0.145 |  |  |
| Obese | 2.47 (0.46; 13.38) | 0.291 | 1.81 (0.96; 3.43) | 0.066 |  |  |
| Lifestyle factors |  |  |  |  |  |  |
| Smoker^g^ | 0.24 (0.03; 1.92) | 0.178 | 0.49 (0.25; 0.98) | 0.045 |  |  |
| Daily alcohol intake^h^ | 0.45 (0.12; 1.66) | 0.228 | 0.54 (0.34; 0.87) | 0.011 |  |  |
| Regular exercise^i^ | 0.84 (0.29; 2.46) | 0.751 | 0.64 (0.38; 1.07) | 0.091 |  |  |
| Multimorbidity^j^ | 4.11 (1.10; 15.27) | 0.035 | 2.37 (1.47; 3.84) | <0.001 |  |  |
| MPL, manageable pain levels; BMI, body mass index; CI, confidence interval; OA, osteoarthritis; UPL, unmanageable pain levels; OR, odds ratio.  ^a^Reference class: male sex; ^b^Reference class: 45-55 years; ^c^Reference class: Lisboa; ^d^Reference class: no partner; ^e^Reference class: <4 years; ^f^Reference class: underweight/normal weight; ^g^Reference class: nonsmoker; ^h^Reference class: no alcohol intake; ^c^Reference class: no regular exercise; ^j^Reference class: no multimorbidity. | | | | | |  |

**Table S.4-** Multivariable models factors associated with UPL in people with Knee OA or Hip OA

|  | Hip OA |  | Knee OA |  |  |  |
| --- | --- | --- | --- | --- | --- | --- |
|  | UPL vs. MPL  OR (95% CI) | p-value | UPL vs. MPL  OR (95% CI) | p-value |  |  |
| Female sex^a^ | 4.46 (1.51; 13.21) | 0.007 | 2.13 (1.40; 3.25) | <0.001 |  |  |
| BMI^b^ | Not included | Not included |  |  |  |  |
| Overweight |  |  | 1.70 (0.92; 3.14) | 0.092 |  |  |
| Obese |  |  | 1.90 (1.01; 3.56) | 0.046 |  |  |
| Lifestyle factors |  |  |  |  |  |  |
| Multimorbidity^c^ | 3.91 (1.22; 12.49) | 0.022 | 2.12 (1.34; 3.36) | 0.001 |  |  |
| *n*^hipOA^=184. Wald F(2)=7.20, p<0.001. *n*^kneeoA^=871. Wald F(4)=7.10, p<0.001  MPL, manageable pain levels; BMI, body mass index; CI, confidence interval; OA, osteoarthritis; UPL, unmanageable pain levels; OR, odds ratio.  ^a^Reference class: male sex; ^b^Reference class: underweight/normal weight; ^c^Reference class: no multimorbidity. | | | | | |  |

**Table S.5.** HOOS/KOOS ADL and QoL subscale scores and anxiety and depression symptoms in people with Hip OA and UPL or MPL and people with Knee OA and UPL or MPL

|  | Hip OA | | |  | | Knee OA | | |  | Hip OA | | | |  | Knee OA | | | |
| --- | --- | --- | --- | --- | --- | --- | --- | --- | --- | --- | --- | --- | --- | --- | --- | --- | --- | --- |
| Sample size and weighted prevalence (%) | **Total**  *n*=184 (100%) | **UPL**  *n*=144 (69.7%) | **MPL**  *n*=40 (30.3%) |  | **Total**  *n*=941 (100%) | | **UPL**  *n*=694 (69.6%) | **MPL**  *n*=247  (30.4%) |  | **β**  **(95% CI)** | **p-value** | **Adjusted^a^ β**  **(95% CI)** | **p-value** |  | **β**  **(95% CI)** | **p-value** | **Adjusted^b^ β**  **(95% CI)** | **p-value** |
| HOOS ADL, mean±SD | 67.25  ±24.46 | 57.78  ±21.19 | 89.09  ±16.21 | **KOOS ADL, mean±SD** | 65.32  ±22.90 | | 58.30  ±21.51 | 81.60  ±16.98 | **UPL** | -31.31  (-39.74, -22.88) | <0.001 | -27.48  (-35.29, -19.67) | <0.001 | UPL | -23.31  (-27.17; -19.44) | <0.001 | -20.01 (-23.65; -16.37) | <0.001 |
| HOOS QoL, mean±SD | 56.48  ±24.36 | 47.46  ±21.51 | 77.12  ±16.89 | **KOOS QoL, mean±SD** | 49.78  ±22.19 | | 42.93  ±19.40 | 65.46  ±20.16 | **UPL** | -29.66  (-37.45; -21.88) | <0.001 | -27.45 (-35.81; -19.08) | <0.001 | UPL | -22.53 (-27.06; -18.00) | <0.001 | -19.83 (-23.99; -15.68) | <0.001 |
|  |  |  |  |  |  | |  |  |  | **Crude OR**  **(95% CI)** | **p-value** | **Adjusted^a^ OR (95% CI)** | **p-value** |  | **Crude OR**  **(95% CI)** | **p-value** | **Adjusted^b^ OR (95% CI)** | **p-value** |
| Anxiety symptoms, n (%) | 30 (15.9) | 29 (22.2) | 1 (1.3) |  | 182 (18.2) | | 146 (19.8) | 36 (14.6) | **UPL** | - | - | - | - | UPL | 1.45 (0.89; 2.34) | 0.132 | 1.12 (0.67; 1.86) | 0.658 |
| Depression symptoms, n (%) | 30 (13.2) | 28 (17.7) | 2 (2.9) |  | 146 (16.6) | | 116 (17.7) | 30 (14.3) | **UPL** | - | - |  | - | UPL | 1.28 (0.65; 2.52) | 0.471 | 0.96 (0.49; 1.90) | 0.914 |
| All percentages and means±SDs are weighted. β^a^ and OR^a^ are adjusted for sex, and multimorbidity .β^b^ and OR^b^ are adjusted for sex, obesity, and multimorbidity.  ADL, activities of daily living; MPL, manageable pain levels; CI, confidence interval; HOOS, Hip Disability and Osteoarthritis Outcome Scale; UPL, unmanageable pain levels; KOOS, Knee Injury and Osteoarthritis Outcome Scale; OR, odds ratio; QoL, quality of life; SD, standard deviation | | | | | | | | | | | | | | | | | | |
